# 血小板源生长因子家族在非小细胞肺癌中的研究进展

**DOI:** 10.3779/j.issn.1009-3419.2014.01.07

**Published:** 2014-01-20

**Authors:** 逸俊 田, 倩 褚, 元 陈

**Affiliations:** 430030 武汉，华中科技大学同济医学院附属同济医院肿瘤科 Department of Oncology, Affiliated Tongji Hospital of Tongji Medical College, Huazhong University of Science and Technology, Wuhan 430030, China

**Keywords:** 血小板源生长因子, 血小板源生长因子受体, 肺肿瘤, 伊马替尼, 舒尼替尼, Platelet derived grow factor, Platelet derived grow factor receptor, Lung neoplasms, Imatinib, Sunitinib

## Abstract

非小细胞肺癌（non-small cell lung cancer, NSCLC）作为全球癌症相关死亡率较高的恶性肿瘤，目前仍缺少可靠稳定的预后指标。血小板源生长因子（platelet derived grow factor, PDGF）及其受体通过多种细胞内信号通路参与细胞生长，迁移，转移以及上皮间叶转化等过程。病理结果表明，PDGF通路主要通过旁分泌途径刺激NSCLC肿瘤间质生长，亦有发现PDGF通路对某些NSCLC肿瘤细胞可能存在直接驱动作用。NSCLC组织中的PDGF及其受体的表达与肿瘤的预后，淋巴结转移等临床结果相关。在临床治疗中，PDGF通路对NSCLC血管生成的重要作用，及抑制PDGF通路促进化疗药物在实体瘤内部分布的作用不容忽视。PDGF作为重要的促血管生成通路，在NSCLC放射治疗中的作用也越来越多地被各种基础研究证实。本文拟对PDGF通路在NSCLC领域的研究进展做一综述，以求对临床和基础研究者有一些启发。

## Abstract (en)

Non-small cell lung cancer (NSCLC) is a malignant tumour with quite high cancer specific mortality, and it still lacks stable and reliable markers for NSCLC' s prognosis. Platelet derived grow factor (PDGF) and PDGFR has been considered to be involved in the process of cell proliferation, migration, metastasis and epithelial mesenchymal transition of cancer cell through various intracellular signal pathways. Pathology analysis showed that PDGF pathway mainly stimulates the proliferation of NSCLC tumour stroma through paracrine pattern, and some reaseach found that PDGF pathway directly promotes some NSCLC cell's proliferation. The expression of PDGF and PDGFR within NSCLC tissue correlates with status of lymphatic metastasis and patients' prognosis. In clinical treatment of NSCLC, the great effect of PDGF pathway in angiogenesis and promoting distribution of chemotherapy by inhibition of PDGF should not be neglected. As an important pro-angiogenesis pathway, functions of PDGF in radiotherapy is dicovered by more and more fundamental research. This review focuses on the progress of PDGF pathway in NSCLC and aims to provide some new ideas for clinical and fundamental researchers.

非小细胞肺癌（non-small cell lung cancer, NSCLC）作为全球癌症相关死亡率最高的恶性肿瘤之一，其疗效预测指标和预后因子尚未明确。以各种肿瘤信号通路为靶点的靶向治疗在NSCLC的综合治疗中发挥了越来越重要的作用，其中抗肿瘤血管生成药物（贝伐珠单抗、血管内皮抑素）在临床应用中所展现出来的作用有目共睹。血小板源生长因子（platelet derived grow factor, PDGF）作为重要的促血管生成通路，其作用也越来越多地被人们了解。本文将对PDGF通路在NSCLC的诊断、治疗以及预后等方面的研究进展进行综述。

## 1 PDGF与肿瘤信号转导通路

### 1.1 PDGF及其受体

PDGF由四种多肽链A、B、C、D以二硫键形成二聚体结构，即可形成PDGF-AA、PDGF-BB、PDGF-CC、PDGF-DD，此外，A链和B链还可形成异源的二聚体，即PDGF-AB，因此目前发现的PDGF有五种二聚体形式^[1]^编码以上四条肽链的基因分别位于染色体7q22、22q13、4q31和11q22^[2]^。血小板源生长因子受体（PDGFR）包括亚单位α和β，每个亚单位由胞外配体结合区，跨胞膜区和胞内酪氨酸激酶活性结构区组成。正常状态下PDGFR亚单位以单体或疏松结合二聚化形式存在于胞膜表面，PDGF与PDGFR结合时，两种亚单位连结为稳定二聚化形式（αα、αβ、ββ），进而发生磷酸化反应，产生生理效应，α亚单位和PDGF的A、B、C链亲和力较高，β亚单位和PDGF的B、D链亲和力较高^[3]^。

### 1.2 PDGF涉及多种细胞内信号转导通路

分泌形式的PDGF与靶细胞表面PDGFR结合以后，使PDGFR发生二聚化，进而激活受体的胞内酪氨酸位点磷酸化，磷酸化的酪氨酸位点又与胞内某些信号分子通路的SH2结构域相互作用，使这些通路被激活。上述胞内信号分子包括：酪氨酸激酶Src亚族、磷脂酶C（PLC-γ）、磷脂酰肌醇-3激酶（PI3-K）、GTP酶活化蛋白（RAS-GAP）、生长因子结合蛋白（Grb2）、酪氨酸特异磷酸酶（Syp）、Src同源物与交联蛋白（Shc）、适应蛋白（Crk）以及非受体酪氨酸激酶家族（Src）等^[2]^。这些信号分子介导胞质内复杂的信号网络，进而作用于细胞核膜上丝苏氨酸残基位点，导致多种基因调节蛋白磷酸化，影响转录水平的基因表达，最终发挥促细胞生长、迁移、自我修复^[3]^以及上皮间叶转化^[4]^等生物效应。PDGF和PDGFR结合方式及其下游通路见图 1。

**1:**
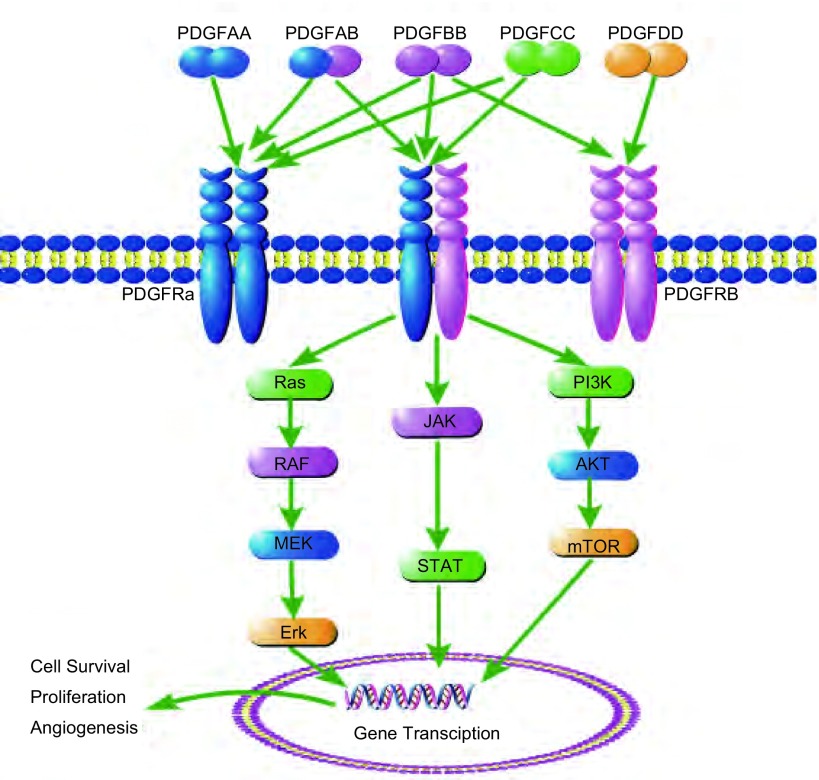
PDGF及其受体结合方式，以及下游主要信号通路示意图 Binding pattern of PDGF, PDGFR and the main signal pathway in the downstream. PDGF: platelet derived grow factor.

## 2 PDGF与NSCLC

### 2.1 PDGF及其受体在NSCLC中的表达

PDGF通路在神经胶质瘤中的自分泌和旁分泌环途径最早被Hermanson等^[5]^发现。此后Vignaud等^[6]^对64例病理确诊为NSCLC患者肺癌组织与8例非肿瘤病变患者的肺组织进行了病理分析对比：发现约1/3的NSCLC患者肺癌细胞PDGF表达增高，1/2的NSCLC患者肺癌细胞PDGFR表达增高，仅有1/6的NSCLC患者肺癌细胞同时有PDGF及其受体的共同增高；大部分NSCLC患者的肿瘤相关巨噬细胞（tumor-associated macrophage, TAM）都有PDGF的表达增高，且TAM多分布于瘤体的外围区域；间叶细胞和内皮细胞在病例组和对照组中都有PDGFR的表达，但无因子的表达；肿瘤细胞和间叶细胞的DNA复制速度加快。由此Jean等认为NSCLC中由肿瘤相关巨噬细胞分泌的PDGF虽然直接刺激肿瘤细胞生长的作用有限，但对肿瘤间质的旁分泌刺激作用更有力。另外，Tsao等^[7]^在肉瘤样NSCLC与对照组NSCLC的免疫组化对比中发现，肉瘤样NSCLC具有更高的PDGFR-β的拷贝数和免疫组化计分。由上述病理结果我们可认为，肉瘤样NSCLC中PDGFR的高表达与PDGF主要调节间质细胞增殖的功能是一致的。肿瘤间质是肿瘤细胞稳定存活于宿主的直接物质基础，由非肿瘤组织，如肿瘤血管、炎性细胞、成纤维细胞、胞外基质等构成，为肿瘤细胞提供血液供应、基质骨架，同时也是机体和肿瘤发生免疫反应的场所^[8]^。PDGF通路通过上述多种胞内促增殖信号促进肿瘤间质，尤其是肿瘤周围血管和淋巴管的生长，从而对肿瘤细胞的生长起促进作用^[9, 10]^。

### 2.2 PDGF及其受体对NSCLC预后的影响

影响肺癌预后的因素多种多样，Andersen等^[11]^对于接受术后放疗的患者肿瘤组织促血管标志物和预后的关系进行了研究，发现在多因素分析中，肿瘤细胞表达PDGF是独立的不良预后因素。Donnem等^[12]^对NSCLC肿瘤细胞和间质细胞（包括内皮细胞、免疫细胞、成纤维细胞等）中PDGF及其受体和疾病相关生存率进行分析发现，NSCLC肿瘤细胞中PDGF-B和PDGFR-β的高表达和不良预后相关，而肿瘤间质中PDGF-A的高表达则和良好预后相关，提示间质细胞中的这种现象可能与高水平PDGF-A能在某种程度上激活适应性免疫有关。Donnem等^[13]^也发现不同的促血管生成通路在不同分期肿瘤的进展和转移中所起的作用并不相同，即肿瘤细胞中VEGF-A/VEGFR-2和VEGFR-3跟T2a期（3 cm＜Φ≤5 cm）NSCLC患者预后有关，PDGF-B跟T2b期（5 cm＜Φ≤7 cm）NSCLC患者预后有关。在淋巴结转移方面，肿瘤细胞PDGF-B的表达和淋巴转移的关系尚存在争议：Cao等^[14]^发现PDGF-BB在体内能有效促进肿瘤淋巴管的生成，使表达PDGF-BB的鼠类纤维肉瘤淋巴管生成增加，且淋巴结转移增多，张楠^[15]^、林文俐^[16]^等的研究也支持了Cao的结论。而Donnem等^[17]^则发现肿瘤细胞PDGF-AA的表达和淋巴结转移相关，和预后关系不明确，PDGF-B（PDGF-BB或PDGF-AB）和VEGFR-3的共表达和不良预后有关，和淋巴转移的关系不明确。郑庆峰等^[18]^通过对205例NSCLC患者手术标本进行免疫组化检测，认为PDGFR与VEGFR-3可能存在某种内在联系。吴丽娜等^[19]^则发现PDGF-B/PDGFR-α自分泌刺激环可能在NSCLC的发生中起重要作用，且阳性表达与疾病的分期呈正相关。

### 2.3 PDGF通路与肺癌治疗

#### 2.3.1 靶向治疗

在对733例NSCLC肿瘤样本的染色体分析中，Ramos等^[20]^发现其中31例样本的染色体4q12有频繁的扩增，且4q12片段中一个长约600 kb的区域明显地增加，此区域基因负责编码PDGFRA及与其紧密相关的受体酪氨酸激酶Kit；体外实验^[21]^对六种肺癌细胞系进行了研究，发现NCI-H1703肺鳞癌细胞系高表达PDGFRA，并且对sunitinib和imatinib两种药物的单药治疗敏感，而sunitinib和imatinib两种靶向药物都对PDGF通路有抑制作用。这些结果表明，NSCLC中存在PDGF通路活化的现象，虽然NSCLC表达PDGF通路的比率并不高，但鳞癌细胞系NCI-H1703对于PDGF通路的依赖性提示其在鳞癌中可能存在的驱动作用。在临床方面，以PDGF通路为靶点之一的小分子酪氨酸激酶抑制剂sunitinib单药在晚期NSCLC中的抗瘤活性已得到证实，Socinski等^[22]^在一项多中心Ⅱ期临床试验中，使用sunitinib单药（4/2 50 mg qd）治疗63例铂类治疗失败的晚期NSCLC患者，部分缓解率为11.1%，中位无进展生存时间为12.0周，中位总生存时间为23.4周。Scagliotti等^[23]^主持的一项Ⅲ期临床试验则比较了sunitinib联合厄洛替尼的方案和厄洛替尼单药在960例复发的晚期NSCLC患者中的疗效差别，证明虽然联合方案没有提高总生存时间，但却能延长无进展生存时间和提高反应率。结合Ramos的研究和上述单药治疗晚期NSCLC患者的临床试验数据，虽然PDGF通路在NSCLC中的突变发生率较低，但在临床研究中，以PDGF为靶点的分子靶向治疗仍在NSCLC的治疗中占有一席之地；同时也必须认识到sunitinib的抗肿瘤血管生成活性并非完全由抑制PDGF通路产生，即VEGF仍然是目前抗血管治疗的重要靶点（表 1）。

**1:** Sunitinib单药以及联合靶向药物治疗NSCLC临床试验汇总 The clinical trials of sunitinib administrated alone or in combined with TKIs in NSCLC

First author	Year	Phase	Criteria	*n*	Regimen	RR (95%CI or *P*)	mPFS (95%CI or *P*)	mOS (95%CI or *P*)
Mark A. Socinski^[22]^	2008	Ⅱ	Ⅲb, Ⅳ, recurrence	63	sunitinib 50 mg/d 4/2	11.1 (4.6-21.6)	12 (10.0-16.1)	23.4 (17.0-28.3)
Silvia Novello^[37]^	2009	Ⅱ	Ⅲb, Ⅳ, recurrence	47	sunitinib 37.5 mg/d CD	2.1 (0.1-11.3)	11.9 (8.6-14.1)	37.1 (31.1-69.7)
Radj Gervais^[38]^	2011	Ⅱ	Ⅲb, Ⅳ	66	sunitinib 50 mg/d 4/2	4/66^a^	15.8 (10.5-21.9)	34.8 (27.2-50.8)
Silvia Novello^[39]^	2011	Ⅱ	Brain metastasis	64	sunitinib 37.5 mg/d CD	1.6 (0-8.8)	9.4 (7.5-13.1)	25.1 (13.4-35.5)
Gu Ping^[40]^	2011	Ⅱ	Ⅲb, Ⅳ, recurrence	22	sunitinib 50 mg/d 4/2	9.0 (0.1-20.9)	11.8 (8.2-15.3)	23.4 (14.7-32.1)
Shannon E.O’Mahar^[41]^	2011	Ⅱ	Advanced non-squamous cancer	11	erlotinib+sunitinib CD	1/11^b^	-	37.2 (6.8-95.6)
George R. Blumenschein Jr^[42]^	2012	Ⅱ	Ⅲb, Ⅳ, recurrence	30	erlotinib+sunitinib CD	3/30^c^	-	-
Giorgio V. Scagliotti^[23]^	2012	Ⅲd	Ⅲb, Ⅳ, recurrence	960	erlotinib±sunitinib CD	10.6 *vs* 6.9 (0.047)	14.4 *vs* 8.0 (0.002)	36.0 *vs* 34.0 (0.139)
H.J.M. Groen^[43]^	2013	Ⅱd	Ⅲb, Ⅳ, recurrence	132	erlotinib±sunitinib CD	4.6 *vs* 3.0 (-)	11.2 *vs* 8.0 (0.321)	32.8 *vs* 30.4 (0.617)
4/2: Four consecutive weeks for once daily treatment, followed by two weeks of no treatment. CD: continuous daily dosing with no interval. a: one in sixty-six achieved CR, three in sixty-six achieved PR. b: one in eleven achieved PR, which last for sixteen point three months three in thirty achieved PR. c: three in thirty achieved PR. -: data was not available. NSCLC: non-small cell lung cancer; mPFS: median progression-free survival; mOS: median overall survival. RR means response rate which is express in percentage, and the unit of survival time (median progression-free survival and median overall survival) is week.								

#### 2.3.2 化疗

肿瘤间质内液压（interstitial fluid pressure, IFP）指实体肿瘤内部组织和液体共同产生的通常高于正常组织的压强。升高的IFP影响化疗药物向肿瘤内部的运输过程。PDGF通路在调控IFP过程中起着重要作用。Kristian Pietras等^[24]^发现imatinib在鼠类肿瘤模型中增强了泰素和5-Fu的抗肿瘤作用。Bertino等^[25]^在鼠类移植物的实验中证明imatinib在体外以及体内均能增加吉西他滨对于恶性间皮瘤的作用，联合治疗较单药能明显抑制肿瘤生长、延长荷瘤鼠的生存时间。Vlahovic等^[26]^在NSCLC的移植瘤实验中，发现imatinib能增加肿瘤中脂质体阿霉素的浓度，并且能增强多西他赛的抑瘤作用。Zhang等^[27]^的研究则发现在体外对肺腺癌细胞系A549使用imatinib单药处理可以产生剂量依赖的细胞生长抑制，且imatinib可通过抑制PDGFR-α的磷酸化增强顺铂对A549细胞的杀伤作用。虽然基础研究中观察到抑制PDGF通路对化疗所产生的获益，但是PDGF抑制剂在为数不多的临床试验中的表现却不令人满意。Tsao等^[28]^的一项Ⅱ期临床试验，入选了22例有转移的NSCLC患者和7例化疗耐药的头颈鳞状细胞癌患者，均给予多西他赛（每3周1次，60 mg/m^2^）和imatinib（每天400 mg）的治疗，两部分试验均在早期因疗效差或明显的毒副作用而提前关闭，Tsao认为泰素类药物和imatinib之间可能存在潜在的拮抗关系，认为今后对于联合紫杉醇类药物和imatinib的方案（尤其是同时给药方案）的研究应该更加谨慎。Bauman等^[29]^对34例70岁以上晚期NSCLC患者给予泰素（90 mg/m^2^ d3, 10, 17 Q28d）和脉冲剂量imatinib（围绕每次泰素注射前后，即600 mg/d d1-4, 8-11, 14-18）的治疗，达到了32%的总有效率，但中位无进展生存时间和中位总生存时间无改善。Camidge等^[30]^在sunitinib联合培美曲塞加顺铂方案治疗晚期恶性实体瘤的Ⅰ期试验中，发现给予两周sunitinib（37.5 mg, qd），间歇1周（2/1组），并每3周行培美曲塞（500 mg/m^2^）加顺铂（75 mg/m^2^）化疗的方案达到了最大耐受剂量，且使用中因累积的骨髓毒性仍需减少剂量，试验中NSCLC患者所能达到的最好的疗效为稳定。Chow等^[31]^也进行了一项sunitinib联合培美曲塞治疗晚期实体瘤的临床试验，由于去掉了顺铂这种药物，每日持续37.5 mg sunitinib可被患者耐受。在21例NSCLC患者中，有4人获得了部分有效的结果。Reck等^[32]^则在GP（吉西他滨1, 000 mg/m^2^，顺铂80 mg/m^2^）方案中联合sunitinib治疗28例NSCLC患者，可评估的20例患者中共有5例达到部分有效的治疗效果。这些试验提示我们，要从两方面认识PDGF通路在抗肿瘤治疗的作用：一方面要承认抑制PDGF通路所产生的明显减低IFP的作用，改善了化疗药物分布，从而提高对肿瘤细胞的杀伤，另一方面也要注意到正常组织也会因此接受到更大剂量的化疗药物，导致毒性的累积。只有充分了解患者肿瘤细胞及其间质PDGF及其受体的表达，探索更合理的给药方法，才可能使治疗效应提升，毒副作用降低（表 2）。

**2:** Imatinib及sunitinib联合化疗治疗NSCLC临床试验汇总表 The clinical trials of sunitinib or imatinib administrated in combined with chemotherapy in NSCLC

First author	Year	Phase	Criteria	*n*	Regimen	RR (95%CI or *P*)	mPFS (95% CI or *P*)	mOS (95% CI or *P*)
Martin Reck^[32]^	2010	Ⅰ	Ⅲb, Ⅳ, no prior treatment	28	GP (gemstabin+cisplatin) 1/2+Sn CD/2/1	20.8 (-)	-	-
Mark A. Socinski^[44]^	2010	Ⅱ	Ⅲb, Ⅳ	56	TCB (paclitaxel+caborplatin+bevacizumab) 1/2±Sn 2/1	8 *vs* 26（-）	15.2 *vs* 18.0（-）	26.4 *vs* -
Chao H. Huang^[45]^	2011	Ⅱ	Relapse or Progress	23	Docetaxel 30 mg/m^2^ 3/1+Im 600 mg/d	1/23^a^	7.6 (-)	24.4 (-)
Anne S. Tsao^[28]^	2011	Ⅱ	metastasis	22	Docetaxel 60 mg/m^2^ 1/2+Im 400 mg/d	1/22^b^	7.9 (-)	35.6 (-)
Julie E. Bauman^[29]^	2012	Ⅱ	Ⅲb, Ⅳ, no prior treatment	34	Paclitaxel 90 mg/m^2^ 3/1+Im 600 mg/d PD	32 (17, 51)	14.4 (-)	29.2 (-)
L. Q. M. Chow^[31]^	2012	Ⅰ	Recurrent or advanced	21	Pemetrexed 1/2+Sn CD/2/1	24 (-)	-	-
D. Ross Camidge^[30]^	2013	Ⅰ	Recurrent or advanced	15	AP (pemetrexed+cisplatin) 1/2+Sn CD/2/1	-	-	-
1/2: One week for chemotherapy treatment, followed by two weeks of no treatment. 2/1: Two consecutive weeks for once daily treatment, followed by two weeks of no treatment. 3/1: Three weeks for weekly paclitaxel therapy, followed by one week of no treatment. CD: continuous daily dosing with no interval. a: one in twenty-three achieved PR. b: one in twenty-one achieved PR. -: data were not available. PD: Pulse dose bracketing each paclitaxel infusion. RR means response rate which is express in percentage, and the unit of survival time (median progression-free survival and median overall survival) is week.								

#### 2.3.3 PDGF通路与肺癌的放疗

Holdhoff等^[33]^在体外实验中发现放疗前给予imatinib处理能增强射线对胶质瘤细胞（RS-1）的杀伤作用，且这种作用主要是由于PDGFR的抑制介导的。D’Amico等^[34]^使用sunitinib处理鼠移植胶质瘤模型后再给予低剂量照射的联合方法，较单独使用两种治疗手段，能更有效抑制肿瘤生长；高剂量照射下加用sunitinib产生严重的毒副反应。尽管这种联合治疗能明显抑制肿瘤的生长，但动物的生存时间并没有改善。Sennino等^[10]^认为在肿瘤细胞中VEGF通路主要控制肿瘤血管密度而不影响周细胞密度，而PDGF通路则涉及募集周细胞而不影响血管密度。周细胞（pericyte）是一类与毛细血管具有密切关系的血管外平滑肌样细胞，参与维持血管密度，血管生成和血管重塑等过程，其募集和分化需要PDGF的调控和参与^[35]^。抑制PDGF通路则会减少周细胞的募集，从而抑制肿瘤血管的自我修复，增强电离辐射对肿瘤的杀伤作用。另一方面，抑制PDGF通路在新奇的放射免疫疗法中也表现出神奇的增效作用。其原理在于拮抗PDGF致肿瘤组织间质液压下降而导致免疫放疗药物更多地被摄入至实体肿瘤细胞，从而增强疗效。Baranowska-Kortylewicz等^[36]^以结肠腺癌细胞系LS174T的裸鼠移植物为对象，发现同时使用免疫放疗药物和STI571较单独使用两者能更有效抑制肿瘤生长，同样的结论在胰腺癌和前列腺癌中重复。由此可见，体外条件下PDGF通路因其独特的修复血管作用及降低肿瘤间质液压作用而使肿瘤细胞对放疗和免疫放疗过程抵抗，抑制这一通路也取得了一定的放疗增效的结果。然而，在动物实验甚至人体试验中，抑制这一通路所产生的副作用，以及在肺癌细胞中的增效作用具体如何，仍需要更多的实验来证实。

## 3 展望

随着抗血管生成类药物（贝伐珠单抗、恩度等）越来越广泛地应用于临床，耐药现象的发生也越来越频繁。抑制VEGF通路这一处于中心地位的血管生成通路，势必导致人体中其它血管生成通路的激活，削弱抑制VEGF所产生的抗血管生成作用。PDGF通路在肺癌发生发展中直接促肿瘤生长、促进肿瘤血管生成的作用，以及PDGF靶向药物单药或联合常规肺癌治疗手段中可能发挥的治疗效应，都需要更多的基础研究和临床试验来阐明，从而为设计新型的抗肿瘤药物提供理论基础。
